# Lesion distribution characteristics of deep infiltrating endometriosis with ovarian endometrioma: an observational clinical study

**DOI:** 10.1186/s12905-020-00974-y

**Published:** 2020-05-20

**Authors:** Hungling Kwok, Hongye Jiang, Tian Li, Huan Yang, Hui Fei, Li Cheng, Shuzhong Yao, Shuqin Chen

**Affiliations:** 1 Department of Obstetrics and Gynecology, the Seventh Affiliated Hospital of Sun Yat-sen University, Shenzhen, China; 2 Department of Obstetrics and Gynecology, the First Affiliated Hospital, Sun Yat-sen University, Guangzhou, 510000 China; 3 Department of Gynecology, Shenzhen Second People’s Hospital/ the First Affiliated Hospital of Shenzhen University Health Science Center, Shenzhen, China

**Keywords:** Ovarian endometrioma, Deep infiltrating endometriosis, Lesion distribution characteristics

## Abstract

**Background:**

To investigate the characteristics of deep infiltrating endometriosis (DIE) lesion distribution when associated with ovarian endometrioma (OEM).

**Methods:**

The present study analyzed retrospective data obtained by the First Affiliated Hospital of Sun Yat-sen University, between June 2008 to June 2016. A total of 304 patients underwent laparoscopic surgery for complete removal of endometriosis by one experienced surgeon, and histological confirmation of OEM associated with DIE was conducted for each patient. Clinical data were recorded for each patient from medical, operative and pathological reports. Patients were then divided into two groups according to unilateral or bilateral OEM. Patients with unilateral OEM were subsequently divided into two subgroups according to OEM location (left- or right-hand side) and the diameter of the OEM (≤50 and > 50 mm). The distribution characteristics of DIE lesions were then compared between the groups.

**Results:**

DIE lesions were widely distributed, 30 anatomical sites were involved. Patients with associated unilateral OEM (*n* = 184 patients) had a significantly increased number of DIE lesions when compared with patients with bilateral OEM (*n* = 120 patients; 2.76 ± 1.52 vs. 2.33 ± 1.34; *P* = 0.006). Compared with bilateral OEM with DIE, there was a higher rate of intestinal (39.1% vs. 18.3%; *P* < 0.01) and vaginal (17.4% vs. 6.7%; *P* < 0.01) infiltration by DIE lesions in unilateral OEM with DIE. The mean number of DIE lesions was not significantly correlated with the location or size of the OEM (2.83 ± 1.56 vs. 2.74 ± 1.53; *P* = 0.678; and 2.65 ± 1.42 vs. 2.80 ± 1.43; *P* = 0.518, respectively). There was no significant difference between the groups with OEM ≤50 mm and > 50 mm.

**Conclusion:**

Lesion distribution characteristics in women diagnosed with histologically proven OEM associated with DIE were frequently multifocal and severe.

## Background

Endometriosis is a common benign diseases of women of childbearing age [1]. According to histology, endometriosis is characterized into three main types: superficial endometriosis, ovarian endometrioma and deep infiltrating endometriosis (DIE) [2]. DIE is an aggressive form of the disease, penetrating to more than 5 mm under the peritoneal surface [3].DIE is associated with infertility and a variable degree of pelvic pain. It is a multifocal disease primarily affecting the posterior area, and frequently involves the uterosacral ligament, uterine rectum pouch and vaginal rectal diaphragm, as well as the bladder, ureter and rectal wall [4].

For patients with multifocal characteristics of DIE, meaningful improvements in clinical symptoms and quality of life are dependent on the radical exeresis of the lesions [5, 6]. When surgical treatment has been recommended, an accurate diagnosis and knowledge of the precise distribution of the extending lesions are required [4]. However, there are currently no adequately sensitive and specific symptoms nor diagnostic tests for the clinical diagnosis of DIE. The perfect solution would be to utilize a preoperative label for the distribution of DIE to generate a precise map of the DIE lesions.

Ovarian endometrioma (OEM) is the most common type of endometriosis, ~ 50% of DIE patients are also diagnosed with OEM [7]. Previous studies have demonstrated that associated OEM is a marker for greater DIE severity. Compared with patients without OEM, patients with OEM have more pelvic and intestinal areas involved by DIE [8, 9]. In case of OEM, severe pelvic pain is significantly associated with DIE [10]. Indeed, Perello et al. [11] created a predictive model to predict DIE in patient with OEM. However, these studies do not refer to the characteristics of the lesion distribution in OEM patients with DIE. Therefore, the present study investigated OEM in order to determine the lesion distribution characteristics of DIE associated with OEM.

## Methods

### Patients

The present retrospective study was approved by the Institutional Review Board of the First Affiliated Hospital of Sun Yat-sen University (Guangzhou, China). All patients provided written informed consent. A total of 304 consecutive patients underwent laparoscopic surgery for complete removal of endometriosis in the First Affiliated Hospital of Sun Yat-sen University, between June 2008 to June 2016; histological confirmation of OEM associated with DIE was conducted for each patient. Histological diagnoses were based on characteristics previously defined in a standard histological description, comprised of a combination of endometrial glands and stroma [12].

The exclusion criteria were as follows: i) laparoscopic surgery converted to open surgery; ii) lack of histological confirmation of endometriosis; iii) previous history of hysterectomy or oophorectomy; and iv) patients with pelvic malignant tumors.

Between June 2008 to June 2016, clinical data were recorded retrospectively for each patient from medical, operative and pathological reports; data were blinded for examination by two of the authors. For each patient, general data were recorded, including age, parity, the diameter and location of the OEM (right or left), and past history of surgical treatment for endometriosis, pain symptoms and other specific symptoms. Pain symptoms included dysmenorrhea, dyspareunia, chronic pelvic pain, and bowel pain. Other specific symptoms including hematochezia, urinary frequency and urgency, and hydro-nephrosis. Histological confirmation was obtained for all endometriotic lesions and a description of the location of the DIE lesions was recorded.

All of the patients underwent extensive preoperative tests, including clinical examinations, transvaginal sonography, urological ultrasound and MRI. Patients were treated surgically due to ineffective conservative treatment or serious complications. All of the operations were performed by an experienced surgeon (Professor Shuzhong Yao). All visible endometriosis lesions, including ovarian endometrioma and DIE lesions, were surgically removed. For patients with adenomyosis, local resection or hysterectomy was performed according to the severity of the disease and bearing requirement. In addition to DIE resection, surgical treatment involves adhesion separation as well as resection and reconstruction of the urinary organs and bowel. When these procedures were employed, urologists and gastroenterologists were necessary. Patients were divided into two groups according to the type OEM: unilateral or bilateral. Patients with unilateral OEM were subsequently divided into subgroups according to the location of the OEM (left- or right-hand side) and the diameter of the OEM (≤50 and > 50 mm). The distribution characteristics of DIE lesions were then compared between the groups.

### Statistical analysis

Statistical analysis was performed using SPSS version 23.0 for Windows (IBM Corp., Armani, NY, USA). *P* < 0.05 was considered to indicate a statistically significant difference. Statistical analysis was conducted stepwise after assessing quantitative variable distributions. Statistical studies using parametric tests were only conducted after checking the normal distribution of the studied variables. Correlations between qualitative variables were analyzed by a Pearson’s chi-square test. In case of significant reciprocal correlation, the relative risk was calculated with 95% confidence intervals.

## Results

Patient characteristics are presented in Table 1. A total of 304 patients were included in the present study. The female patients had a mean age of 34.2 ± 5.8 (range, 17–49) years. All the patients with OEM associated with DIE have different symptoms. According to the different symptoms, they were divided into two types: non-specific symptoms and specific symptoms. Non-specific symptoms include dysmenorrhea (89.5%), dyspareunia (12.8%) and chronic pelvic pain (4.3%), while specific symptoms include intestinal symptoms and urinary symptoms. Intestinal symptoms include rectal tenesmus (24%), difficulty in defecation (4.3%), defecation pain (3.6%), diarrhea (2.0%) and hematochezia (2.6%). Urinary symptoms include urinary frequency and urgency (3.0%), hematuria (0.7%) and hydronephrosis (8.6%). In the cohort of 304 patients with an associated OEM, the OEM was located on the left-hand side in 98 patients (32.2%), on the right-hand side in 86 patients (28.3%), and was bilateral in 120 patients (39.5%). The mean size of the OEM was 55.9 ± 23.9 mm (range, 10–150 mm).

**Table 1:** Preoperative characteristics of the study population

Patients’ characteristics (*N* = 304)	Values ^a^
Age(years)	34.2 ± 5.8(range17–49)
Parity	
Para≥1	158(52.0%)
Nulliparous	146(48.0%)
Infertility	78(25.7%)
Previous surgery for OEM	88(28.9%)
Presenting symptoms^b^	
No symptoms	32(10.5%)
Dysmenorrhea	272(89.5%)
Dyspareunia	39(12.8%)
Chronic pelvic pain	17(5.6%)
Rectal tenesmus	73(24.0%)
Difficulty in defecation	13(4.3%)
Diarrhea	6(2.0%)
Defecation pain	11(3.6%)
Hematochezia	8(2.6%)
Urinary frequency and urgency	9(3.0%)
Haematuria	2(0.7%)
Hydronephrosis	26(8.6%)
Endometrioma laterality	
Left	98(32.2%)
Right	86(28.3%)
Bilateral	120(39.5%)
Endometrioma size (mm)	55.9 ± 23.9 (range10–150)
Number of DIE lesions	2.60 ± 1.5 (range1–9)

OEM, ovarian endometrioma; DIE, deep infiltrating endometriosis

^a^Values are shown as mean ± standard deviation or N(%)

^b^Different symptoms can be associated in the same patient

The anatomical locations of the DIE lesions are presented in Table 2. A total of 788 histologically confirmed DIE lesions were observed in the present study. The mean number of DIE lesions per patient was 2.60 ± 1.46 (range, 1–9). DIE lesions were widely distributed; there were 30 anatomical sites recorded in the present study, which were mainly located in the posterior pelvic cavity. A total of 532 lesions were recorded, of which 274 (51.5%) were located on the left-hand side and 258 (48.5%) on the right-hand side. The uterosacral ligament represented the most frequent location site with a prevalence of 80.6%, followed by the intestine (30.6%), ureter (15.5%), vagina (13.2%), posterior fornix (10.9%), fallopian tube (9.54%), vaginal-rectum (6.91%), rectovaginal pouch (2.96%) and bladder (1.32%). Intestinal endometriosis includes rectum and sigmoid endometriosis. There were also 4 cases of appendiceal endometriosis that were not included in the intestinal endometriosis.

**Table 2:** The prevalence of anatomical distribution of DIE lesions. (n = 304 patients)

Main lesion^a^	Number of patients(%)^b^
Uterosacral ligament	245(80.6)
Left	57
Right	53
Bilateral	135
Intestine	93(30.6)
Ureter	47(15.5)
Left	22
Right	18
Bilateral	7
Vaginal	40(13.2)
Posterior fornix	33(10.9)
Fallopian tube	29(9.54)
Left	12
Right	4
Bilateral	13
Recto-vaginal septum	21(6.91)
Rectovaginal pouch	9(2.96)
Bladder	4(1.32)

*DIE* deep infiltrating endometriosis

^a^According to the location of the lesion recorded during the operation

^b^Number of patients whose lesions histologically proven deep infiltrating endometriosis

Patients with associated unilateral OEM (*n* = 184 patients) had a significantly increased number of DIE lesions when compared with patients with bilateral OEM (*n* = 120 patients; 2.76 ± 1.52 vs. 2.33 ± 1.34; *P* = 0.006; Table 3). Compared with bilateral OEM with DIE, a higher rate of intestinal (39.1% vs. 18.3%; *P* < 0.01) and vaginal (17.4% vs. 6.7%; P < 0.01) infiltration by DIE lesions was observed in unilateral OEM with DIE. The mean number of DIE lesions was not significantly correlated with the location or size of the OEM (2.83 ± 1.56 vs. 2.74 ± 1.53, *P* = 0.678; 2.65 ± 1.42 vs. 2.80 ± 1.43, *P* = 0.518, respectively). There were no significant differences between the groups with OEMs ≤50 mm and > 50 mm.

**Table 3:** Characteristics of deeply infiltrating endometriosis lesion distribution associated ovarian endometrioma

Variables	Comparison		*P*-value	OR	95% CI
	OEM laterality^a^				
	Unilateral *n* = 184(%)	Bilateral *n* = 120(%)			
Number of DIE lesions	2.76 ± 1.52	2.33 ± 1.34	0.006^*^		
Uterosacral ligament	151(82.1)	94(78.3)	0.421	1.266	0.712–2.249
Intestine	72(39.1)	21(17.5)	0.000^*^	3.031	1.738–5.286
Vagina	32(17.4)	8(6.70)	0.007^*^	2.947	1.308–6.641
Posterior fornix	23(12.5)	10(8.3)	0.100	1.571	0.720–3.431
Ureter	29(15.8)	18(15.0)	0.858	1.060	0.560–2.009
Fallopian tube	19(10.3)	10(8.3)	0.563	1.267	0.568–2.827
Rectovaginal pouch	16(8.7)	14(11.7)	0.396	0.767	0.359–1.637
	Side of OEM^a^				
	Left side *n* = 98(%)	Right side *n* = 86(%)			
Number of DIE lesions	2.83 ± 1.56	2.74 ± 1.53	0.678		
Uterosacral ligament	81(82.7)	70(81.4)	0.824	1.089	0.512–2.315
Intestine	40(40.8)	32(37.2)	0.617	1.164	0.642–2.109
Vagina	18(18.4)	15(17.4)	0.870	1.065	0.500–2.268
Posterior fornix	15(15.3)	8(9.3)	0.219	1.762	0.078–4.386
Ureter	16(16.3)	13(15.1)	0.822	1.096	0.494–2.431
Fallopian tube	8(8.2)	11(12.8)	0.303	0.606	0.232–1.584
Rectovaginal pouch	9(9.2)	7(8.1)	0.802	1.141	0.406–3.207
	Unilateral OEM size^a^				
	≤50 mm *n* = 112(%)	> 50 mm *n* = 72(%)			
Number of DIE lesions	2.77 ± 1.4	2.82 ± 1.4	0.960		
Uterosacral ligament	87(77.7)	64(88.9)	0.053	0.435	0.184–1.027
Intestine	54(48.2)	17(23.6)	0.001^*^	3.012	1.560–5.817
Vagina	25(22.3)	6(8.3)	0.013^*^	3.161	1.226–8.164
Posterior fornix	11(9.8)	12(16.7)	0.171	0.545	0.226–1.311
Ureter	15(13.4)	14(19.4)	0.272	0.641	0.289–1.423
Fallopian tube	10(8.9)	9(12.5)	0.437	0.686	0.264–1.781
Rectovaginal pouch	11(9.8)	12(16.7)	0.171	0.545	0.226–1.311

^a^Values are shown as mean ± standard deviation or *N*(%)

^*^Statistically significant

## Discussion

Previous studies have revealed that, for patients with histologically confirmed DIE, an associated ovarian OEM is a marker for the severity of the disease [8]. However, the related research on the distribution characteristics of the DIE lesions in patients with OEM is still limited. The present study investigated lesion distribution characteristics in women diagnosed with histologically confirmed OEM and DIE, as this combined diagnosis is often indicative of a more multifocal and severe disease. There was a greater frequency of anatomic DIE lesion sites in patients with unilateral OEM (unilateral OEM size ≤50 mm) than in patients with bilateral OEM (unilateral OEM size ≤50 mm), and DIE lesions were often associated with intestinal and vaginal infiltration.

These data suggested that DIE lesions were widely distributed. There were 30 anatomical sites observed in the present study, which were primarily located in the posterior pelvic cavity, but also in the uterosacral ligaments, intestines, ureter, vagina, posterior fornix and rectovaginal pouch. The distribution of DIE lesions was associated with the flow pattern of peritoneal fluid and the morphology of the pelvic cavity [13]. When compared with the uterosacral ligaments, intestines and vagina, the reduced frequency of deep bladder endometriosis observed can be explained by anatomical location as the lower limit of the vesico-uterine pouch is located well above the lower limit of the Pouch of Douglas [14]. The anatomical differences between the left- and right-hand sides of the hemipelvis are that the sigmoid colon is located on the left-hand side of the hemipelvis, and when combined with the left adnexa it forms a barrier to prevent menstrual blood reflux, resulting in an anatomical situation that could promote adhesions and the growth of refluxed endometrial cells on the left-hand side of the pelvic wall [15].

According to the literature, intestinal endometriosis and urinary tract endometriosis account for ~ 5–12% and 0.3–12%, respectively, of all women with endometriosis [16–18]. However, in the present study, intestinal endometriosis accounted for 30.9%, and urinary tract endometriosis accounted for 15.2% of cases, which are greater frequencies than those recorded by previous research. According to the theory of Kondo et al., women presenting with OEM had a stronger association with the presence of DIE lesions and intestinal DIE [19]. There is lack of research on OEM associated with urinary tract endometriosis. The present results indicated that, for patients diagnosed with histologically verified OEM associated with DIE, there was an increased risk of lesions involving the intestine and ureter. Thus, when the clinical examination suggests OEM with DIE, the practitioner should search for severe lesions, especially intestinal and ureteral lesions. Surgery for intestinal endometriosis and urinary endometriosis is difficult, with a number of postoperative complications [20, 21]. The literature reports distinguish between major and minor complications. Major complications include anastomotic insufficiencies, intestinal perforation, retovaginal fistulas, severe infections, and bleeding requiring transfusion, which are reported in 7.4% [22] to 25% [23]. Minor complications include slight-to-moderate infections, peripheral sensory disturbances, bladder voiding dysfunction, and postoperative urinary obstruction, which are reported in 0.6% [24] to 57% [25]. Therefore, full clinical evaluations, as aforementioned, before operating may be very important for OEM patients with DIE.

As mentioned above, intestinal endometriosis refers to the rectum and sigmoid endometriosis. However, there are a special intestinal endometriosis, appendiceal endometriosis, which is considered as an uncommon finding, in the literature its prevalence varies widely [26]. In our study, there are 4 patients with appendiceal endometriosis , are responsible for approximately 4% of all intestinal lesions. Mabrouk et al. [27] pointed out that appendiceal endometriosis was associated with adenomyosis, large right endometrioma, deep posterior pelvic endometriosis, left deep lateral pelvic endometriosis, and ileocecal involvement. The 4 patients in our study who had appendiceal endometriosis were all combined with adenomyosis, and the diameter of OEM was greater than 5 cm, which was further validated the conclusions of Mabrouk et al.

Patients with simple OEM often have no typical clinical manifestations, and some patients only find the presence of OEM in routine physical. Previous studies have conducted multivariate regression analysis on the relationship between dysmenorrhea and OEM. The results showed that the severity of dysmenorrhea had nothing to do with the existence of OEM. Intestinal endometriosis and deep pelvic invasive endometriosis were the main related factors of dysmenorrhea [28]. Of the 304 patients in our study, 272 (89.5%) with symptoms of dysmenorrhea, 39 (12.8%) with dyspareunia, and 17 (5.6%) with chronic pelvic pain. Dai et al. reported that 61.6% of patients with OEM but non-DIE had dysmenorrhea, and 10.2% had severe dysmenorrhea [29]. It is suggested that when patients with OEM have obvious pain symptoms, it should be considered that they may be complicated with DIE.

During the early stages of ureteral endometriosis, the clinical symptoms are not typical, and are easily overlooked during surgery. Some researchers have suggested that urinary tract endometriosis may occur more frequently than it is currently thought to [30, 31]. Raimondo D et al. observed that ureteral involvement was always associated with endometriosis in other locations in the pelvis. And the most frequent endometriosis associations with ureteral involvement are ovarian lesions [32]. In this case, when there is an associated OEM, ultrasonic examination of the urogenital system is necessary. Ureteral endometriosis should be considered when B mode ultrasound reveals ureter stenosis and hydronephrosis. Hydroureter and hydronephrosis are the severe forms of ureteral endometriosis. In case of hydronephrosis, renal scintigraphy to evaluate renal function should be needed.

Patients with associated unilateral OEM have an increased number of DIE lesions and have a greater risk of lesions involving the intestine and vagina when compared with patients with bilateral OEM. It was hypothesized that a clinical case of bilateral OEM may be more complex than unilateral OEM, and the distribution of the DIE lesions may be more extensive and deeper in patients with larger OEM. However, some researchers maintain that in patients with OEM ≥30 mm, OEM size was the most influential contributor to the total number of follicles and oocytes retrieved. OEM results in a reduced response to ovarian stimulation, when compared with the response of the contralateral normal ovary in the same individual [33]. The oppression of the ovary was more obvious in bilateral OEM and the response to ovarian stimulation was markedly reduced. In the clinic, patients with unilateral OEM or an OEM with a diameter of ≤50 mm should not be taken lightly, and should be evaluated for the presence of DIE lesions, especially intestinal DIE.

The aim of the present study was to potentially provide the basis for avoiding underestimation of the extent of the DIE lesions. Misunderstanding the severity of DIE lesions is the main reason why it is difficult to completely remove lesions during surgical treatments. Incomplete exeresis of DIE lesions explains the high risk of recurrence; this recurrence is in fact the continued progression of the lesions left behind during previous operations [34, 35]. Repeated surgery is positively associated with increased health care costs and morbidity [36]. Repeated surgery with damage to ovarian reserves is particularly frequent in ovarian endometriosis [37–39]. In addition, the risk of repeated operations is associated with uncertainty regarding surgical outcomes and pain. Therefore, completely excising the DIE lesions during the initial surgery is particularly important.

For the first time, our study analyses the characteristic of DIE lesion distribution in patient with ovarian endometrioma. However, this was a retrospective single center study, results were limited by a lack of random, patient selection, and incomplete data acquisition. The data on the history of previous endometriosis treatment were incomplete, which prevented us from evaluate the effect of previous treatment on the recurrence. A large multicenter prospective trial will be necessary to further assess this lesion distribution characteristic.

## Conclusions

In summary, lesion distribution of DIE associated with OEM was frequently multifocal and severe. For patients with OEM that requires surgical treatment, it cannot be treated only by cystectomy; it should be combined with the patient’s clinical symptoms, and be carefully explored during surgery to avoid the omission of the lesions.

## Data Availability

The datasets used and/or analyzed during the current study are available from the corresponding author on reasonable request.

## Abbreviations

DIE: Deep Infiltrating Endometriosis
OEM: Ovarian Endometrioma
